# Construction of Bone Metastasis-Specific Regulation Network Based on Prognostic Stemness-Related Signatures in Prostate Cancer

**DOI:** 10.1155/2022/8495923

**Published:** 2022-03-29

**Authors:** Juanwei Zhuang, Mingxiao Li, Xinkun Zhang, Shuyuan Xian, Jie Zhang, Huabin Yin, Yifan Liu, Mingxiang Fan, Zhenyu Li, Xiaolong Zhu, Ruoyi Lin, Siqiao Wang, Zhitong Zhou, Chenlu Wei, Penghui Yan, Tong Meng, Runzhi Huang, Zongqiang Huang

**Affiliations:** ^1^Department of Orthopedics, The First Affiliated Hospital of Zhengzhou University, 1 East Jianshe Road, Zhengzhou 450052, China; ^2^Tongji University School of Medicine, 1239 Siping Road, Shanghai 200092, China; ^3^Division of Spine, Department of Orthopedics, Tongji Hospital Affiliated to Tongji University School of Medicine, 389 Xincun Road, Shanghai 200065, China; ^4^Department of Orthopedics, Shanghai General Hospital, School of Medicine, Shanghai Jiaotong University, 100 Haining Road, Shanghai, China; ^5^Shanghai Jiao Tong University School of Medicine, No. 227 South Chongqing Road, Huangpu District, Shanghai 200025, China; ^6^Tongji University Cancer Center, Shanghai Tenth People's Hospital, Tongji University School of Medicine, 301 Yanchang Road, Shanghai 200072, China

## Abstract

**Background:**

We planned to uncover the cancer stemness-related genes (SRGs) in prostate cancer (PCa) and its underlying mechanism in PCa metastasis.

**Methods:**

We acquired the RNA-seq data of 406 patients with PCa from the TCGA database. Based on the mRNA stemness index (mRNAsi) calculated by one-class logistic regression (OCLR) algorithm, SRGs in PCa were extracted by WGCNA. Univariate and multivariate regression analyses were applied to uncover OS-associated SRGs. Gene Set Variation Analysis (GSVA), Gene Set Enrichment Analysis (GSEA), and Pearson's correlation analysis were performed to discover the possible mechanism of PCa metastasis. The significantly correlated transcription factors of OS-associated SRGs were also identified by Pearson's correlation analysis. ChIP-seq was applied to validate the binding relationship of TFs and OS-associated SRGs and spatial transcriptome and single-cell sequencing were performed to uncover the location of key biomarkers expression. Lastly, we explored the specific inhibitors for SRGs using CMap algorithm.

**Results:**

We identified 538 differentially expressed genes (DEGs) between non-metastatic and metastatic PCa. Furthermore, OS-associated SRGs were identified. The Pearson correlation analysis revealed that FOXM1 was significantly correlated with NEIL3 (correlation efficient =0.89, *p* < 0.001) and identified hallmark_E2F_targets as the potential pathway mechanism of NEIL3 promoting PCa metastasis (correlation efficient =0.58, *p* < 0.001). Single-cell sequencing results indicated that FOXM1 regulating NEIL3 may get involved in the antiandrogen resistance of PCa. Rottlerin was discovered to be a potential target drug for PCa.

**Conclusion:**

We constructed a regulatory network based on SRGs associated with PCa metastasis and explored possible mechanism.

## 1. Introduction

Prostate cancer is the second prevailing malignant tumor occurred in adult male, which accounted for 13.5% in male malignancies [1].What is worse, the global incidence of prostate cancer is surging continuously, making it one of the most significant causes of the death of male patients with cancer. It is estimated that there were approximately 1.3 million new cases and 0.36 million death cases in 2018 around the world [2, 3]. Prostate adenocarcinoma (PRAD) is the most common type of prostate cancer, originating from glandular epithelial cells. Traditional therapies, including radical prostatectomy, active surveillance, and radiotherapy, often induce patients' depression and anxiety and cause urinary symptoms and sexual and bowel dysfunction, which are quite common in clinical practice [4]. Metastatic PCa is the leading cause of PCa-associated death and mostly occurs in the bone, resulting in severe pain, frequent fractures, and hypercalcemia [5, 6]. Patients with higher Gleason grade, prostate-specific antigen amount, and certain signatures of MRI images should be attached more attention of prostate cancer metastasis in the period of active surveillance [7]. Nowadays, androgen deprivation therapy is recommended for symptomatic patients in M1 phase as first-line treatment to attenuate symptom and reduce risk of suffering severe sequelae caused by terminal illness [8]. In metastatic castration-resistant prostate cancer (mCRPC), taxane therapy could prolong overall survival significantly in patients with Androgen receptor variant 7 (AR-V7), which is a potential marker of treatment selection [5, 6, 8]. There is growing evidence indicating that certain molecular targets may play important roles in prostate cancer metastasis and help to guide treatment options and predict prognosis.

Cancer stem cells (CSCs) are a group of cells that have the ability to self-renew and produce heterogeneous tumor cells. Despite the rareness of CSC in tumor cells, it is regarded as the basis of tumorigenesis, progression, recurrence, and metastasis [9, 10]. CSC has the ability to initiate and maintain cancer cell reproduction, which provides the necessary condition for the eventual formation of metastasis. In addition, the CSC subpopulations with metastatic ability in heterogeneous CSCs could migrate to other parts of the body and form distant metastasis [11, 12]. In order to evaluate how identical tumor cells are to CSCs, researchers developed a new measurement—stemness indices, including mDNAsi and mRNAsi by far. The index ranges from 0 to 1, and the larger index indicates the lower degree of cell differentiation and the higher similarity of stem cells [13, 14]. In our study, we applied mRNA stemness indices (mRNAsi) to assess tumor development. Besides, we identified stemness-related genes (SRGs) in PCa, aiming to discover how these SRGs promote PCa formation, progression, and metastasis.

In our study, we identified 538 differentially expressed genes (DEGs) between non-metastatic and metastatic PCa and calculated mRNA stemness index (mRNAsi) of 406 PCa samples in TCGA database. Then, we applied weighted Gene Co-expression Analysis (WGCNA) to uncover the gene module correlated with mRNAsi and extracted prognostic stemness-related genes (SRGs) in the module by univariate and multivariate Cox regression analyses. The regulatory transcription factor of the OS-associated SRGs was also uncovered by Pearson's correlation analysis, further validated by ChIP-seq. In order to explore the downstream mechanism, we performed Gene Set Enrichment Analysis (GSEV) and Gene Set Variation Analysis (GSVA), and Pearson's correlation analysis. Besides, we applied spatial transcriptome and single-cell sequencing to identify the location of key biomarkers expression. Lastly, we searched for the possible target inhibitors in the Connectivity Map (CMap) database, aiming to discover effective drugs for PCa.

## 2. Method

### 2.1. Data Collection and DEGs Identification

The Ethics Committee of the First Affiliated Hospital of Zhengzhou University approved this study. The clinical information and RNA-seq data of 406 patients with PCa were acquired from the Cancer Genome Atlas (TCGA) Database (https://tcgadata.nci.nih.gov/tcga/). Then, the differentially expressed genes (DEGs) among patients with localized and metastatic PCa were identified, which all satisfy the criterion that FDR <0.05 and |log2 FC| >1. The heat map and volcano plot were generated to illustrate these DEGs, respectively. Furthermore, Gene Ontology (GO) and Kyoto Encyclopedia of Genes and Genomes (KEGG) enrichment analyses were applied to explore the underlying mechanisms of these DEGs.

### 2.2. mRNA Stemness Index Evaluation

The mRNA stem cell index (mRNAsi) is applied to evaluate the activity of cancer stem cells in tumor tissue. It ranges from 0 to 1 and the higher mRNAsi represents a higher dedifferentiation of cancer cells. The mRNAsi of each sample was calculated based on the one-class logistic regression (OCLR) machine-learning algorithm [14], which was shown in the heat map.

### 2.3. Identification of Stemness-Related Genes by Weighted Gene Correlation Network Analysis

In order to further explore the stemness-related genes (SRGs) and its possible mechanisms, weighted Gene Co-expression Analysis (WGCNA) was performed [15]. The identified DEGs were the input data and mRNAsi and expression of hallmarks of cancer was used as clinical phenotype for the co-expression analysis. The cutoff was set to extract SRGs in the module, which required the expression of a single gene was co-expressed significantly with the Egen of module (|Co-expression Coefficient| >0.50) and mRNAsi (|Co-expression Coefficient| >0.50).

### 2.4. Identification of OS-Associated SRGs and Construction of Prognostic Model

In order to evaluate the prognostic value of these SRGs, the univariate Cox regression analysis was applied to test the association between the expression of SRGs and overall survival. SRGs with *p* value <0.001 in univariate Cox regression analysis were defined as OS-related SRGs. Then, the multivariate Cox regression analysis was operated to evaluate the regression coefficient (*β* value) of overall survival associated SRGs. Then, based on the formula (Risk Score =∑_*i*=1_^*n*^*βi* × gene *i*), a prognostic model was constructed to help predict the prognosis of patients with PCa. In the formula, “*n*” represented prognostic SRGs, and “*β*” represented the coefficient of each prognostic SRGs.

In order to test the accuracy of the model, area under ROC (AUC) curve was generated. Then, PCa patients were divided into two subgroups according to the median risk score. The Kaplan-Meier survival analysis was performed to test the difference of survival between the high- and low-risk subgroups. Besides, PCa patients were reordered by the risk score to generate risk curve, scatterplot.

The univariate and multivariate Cox regression analyses were applied to test the independency of the risk score as a prognostic factor, as well as age, Gleason score, and bone metastasis.

### 2.5. Identification of the Potential Pathway Mechanism

In order to explore the underlying pathway mechanism in PCa progression, GSEA was performed to assess the expression of hallmarks of cancer in PCa. In addition, GSVA was also used to screen the significantly differentially expressed hallmarks of cancer between non-metastatic and metastatic PCa (FDR <0.05). What's more, then, the Venn plot was generated to illustrate the intersection of identified hallmarks of cancer in GSEA, GSVA, and co-expressed hallmarks of cancer in WGCNA, which were considered the potential downstream pathway.

### 2.6. The Regulation Pair between Transcription Factors and SRGs

318 cancer-associated transcription factors (TFs) were downloaded from the Cistrome Cancer database (http://cistrome.org/) [16]. There were 33 cancer-associated TFs differentially expressed between non-metastatic and metastatic PCa. Then, Pearson's correlation analysis was operated to uncover the strong correlation pair between differentially expressed cancer-associated TFs and SRGs, whose correlation coefficient >0.300, and *p* < 0.001. Besides, we acquired the ChIP-seq result to verify the direct binding relationship between key TFs and OS-associated SRGs from Cistrome Data Browser [16].

### 2.7. Spatial Transcriptome and Single-Cell Sequencing

SpatialDB is the first single-cell spatial transcriptome data visualization platform, providing the spatial information of gene expression in specific tissue [17]. We downloaded the spatial transcriptome sequencing results of key biomarkers from SpatialDB to detect the location of their expression in prostate cancer tissue. Single Cell Expression Atlas [18] and scRNASeqDB [19] were two online databases for comprehensive transcriptome in a single cell. We identified the exact cell type where the biomarkers in our study were mainly expressed based on single-cell sequencing results in these two databases.

### 2.8. Identification of Candidate Target Drugs

We utilized Connectivity Map (CMap) algorithm to search for possible target drugs for PCa, which might inhibit SRGs and suppress CSCs activity [20]. DEG analysis results of SRGs and TFs were applied as the input data of CMap. Besides, we included the DEG analysis results between high mRNAsi and low mRNAsi group in pan cancer. Finally, specific inhibitors with significance inn over 10 types of cancers were depicted in the heat map. We also acquired the chemical structure of these inhibitors from clue database.

### 2.9. External Databases Validation

In order to further validate the prognostic value of key biomarkers and decrease the bias, we applied multiple online databases to verify the expression of TFs, SRGs, and signaling pathways at gene and protein level, including UALCAN [21], cBioPortal [22], GEPIA [23], Oncomine [24], the Human Protein Atlas [25], UCSC Xena [26], CCLE [27], friebrowse [28], Pathcards [29], and String database [30].

### 2.10. Statistical Analysis

All statistical analysis was performed by R version 3.5.1 (Institute for Statistics and Mathematics, Vienna, Austria; https://www.r-project.org). For descriptive statistics, the continuous variables in normal distribution were described by mean ± standard deviation, while the continuous variables in abnormal distribution were described by median (range). Counts and percentages were applied for categorical variables. Two-tailed *p* < 0.05 was considered statistically significant.

## 3. Results

### 3.1. Identification and Functional Enrichment Analysis of Significantly Differentially Expressed Genes

**Figure 1:**
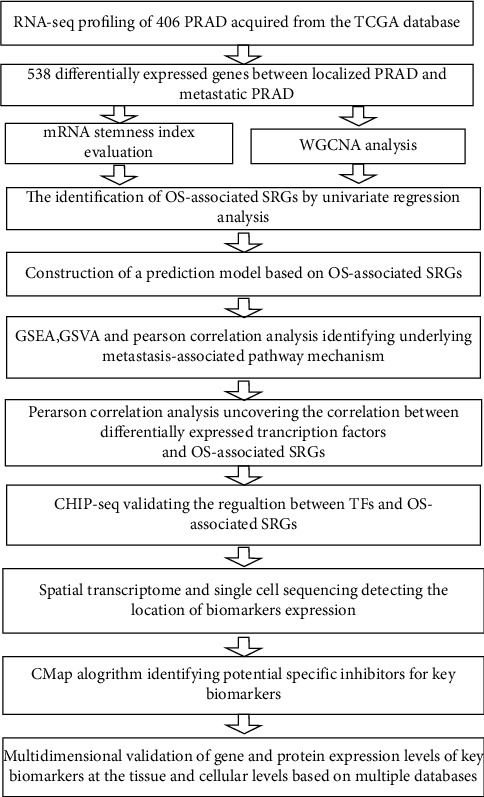
The flowchart of analytical process.

**Figure 2:**
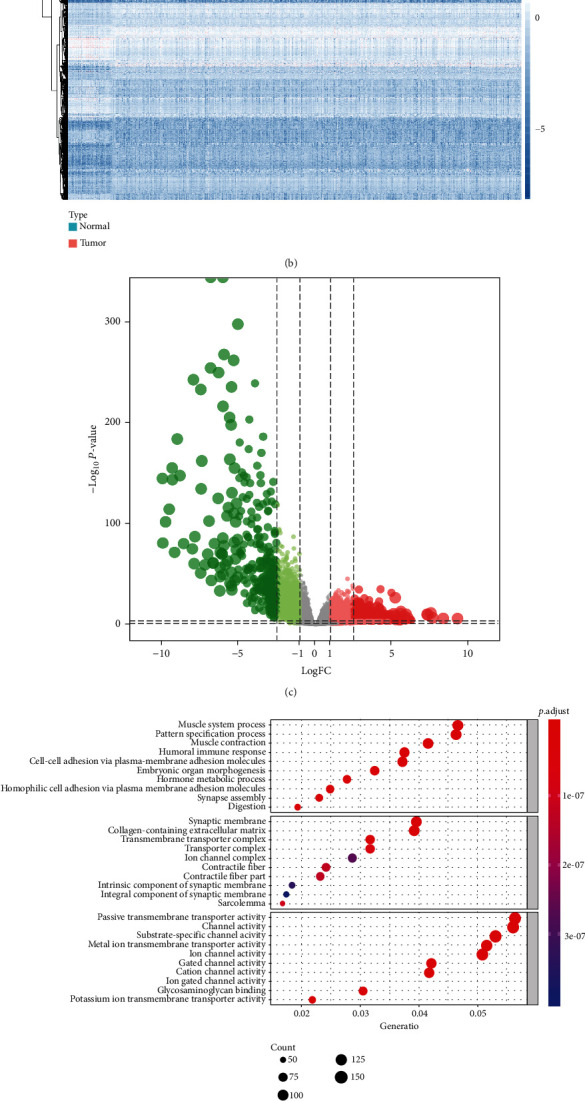
The identification and functional enrichment analysis of differentially expressed genes between localized and metastatic PCa. The heat map of mRNAsi of PCa samples in TCGA database (a). The heat map (b) and volcano plot (c) of significantly differentially expressed genes between localized PCa and metastatic PCa. GO (d) and KEGG (e) enrichment analysis of significantly differentially expressed genes. mRNAsi: mRNA stemness index; GO: Gene Ontology; KEGG: Kyoto Encyclopedia of Genes and Genomes; PCa: prostate cancer.

The overall analytical process is revealed in Figure 1. The baseline clinical information of 406 PCa patients obtained from the TCGA database is represented in Table S1. A total of 538 differentially expressed genes (DEGs) between non-metastatic and metastatic PCa were singled out by the Wilcoxon test, including 10 downregulated genes and 528 upregulated genes in metastatic PCa. All the identified DEGs reached the criterion that the log (fold change) >1 or< −1 and FDR<0.05, illustrated in the heat map and volcano plot (Figures 2(b) and 2(c)). Then, GO and KEGG enrichment analyses were applied to reveal the possible biological mechanism. GO analysis revealed that these DEGs got involved in several biological processes (BP) (including muscle system process and pattern specification process), cellular components (CC) (including synaptic membrane and collagen-containing extracellular matrix), and molecular functions (MF) (including passive transmembrane transporter activity and channel activity) (Figure 2(d)). Meanwhile, KEGG analysis indicated that these DEGs were also related to a plenty of signaling pathways, including neuroactive ligand-receptor interaction, cAMP signaling pathway, and calcium signaling pathway (Figure 2(e)).

### 3.2. Weighted Gene Correlation Network Analysis

**Figure 3:**
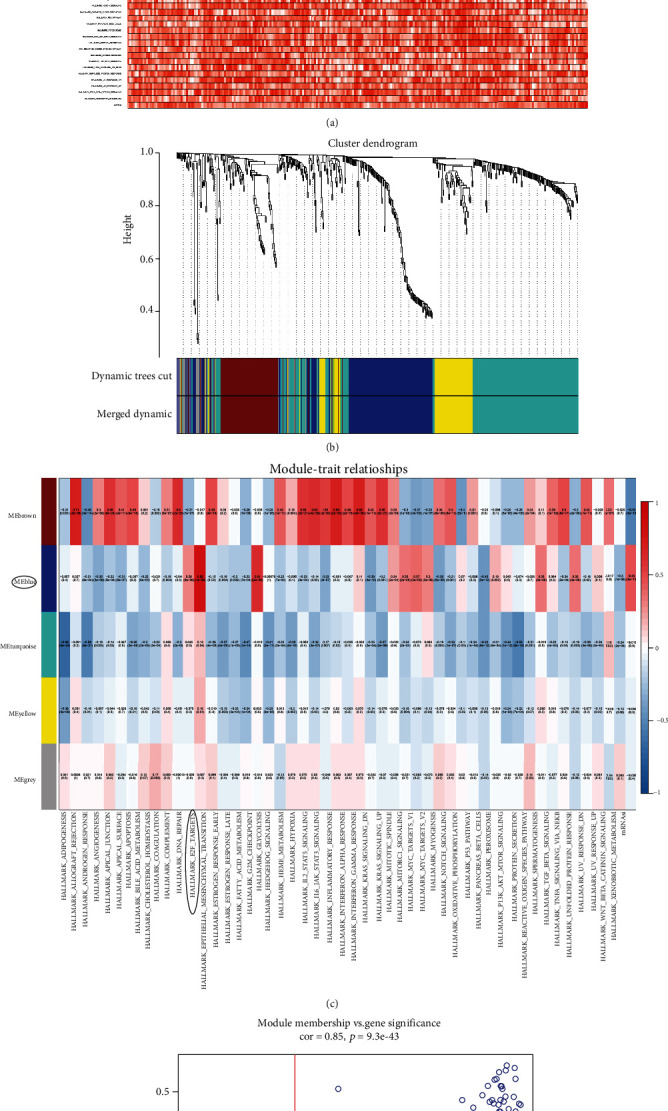
The identification of stemness-associated genes (SRGs) by WGCNA analysis. The sample dendrogram and trait heat map of WGCNA analysis (a). The cluster dendrogram of genes, with dissimilarity based on ontological overlap, as well as merged module colors (b). The heat map revealed that MEblue module had the strongest correlation with mRNAsi, whose most relevant hallmarks pf cancer were hallmark_E2F_targets (Figure 2(C)). The scatter plot filtered genes in MEblue whose module membership >0.3 and gene significance for mRNAsi >0.3.

The heat map revealed the mRNA stem cell index (mRNAsi) of each PCa sample, which was calculated by OCLR machine-learning algorithm (Figure 3(a)) Then, using GSVA method, the expression of hallmarks of cancer in each PCa sample was also calculated. Based on the mRNAsi, expression of hallmarks of cancer, and expression of DEGs, WGCNA was performed to explore the crucial gene models in PCa development and possible mechanisms (Figures 3(a) and 3(b)). The heat map revealed that MEblue module had the strongest correlation with mRNAsi, whose most relevant hallmarks of cancer were hallmark_E2F_targets (Figure 3(c)). The scatter plot screened genes in MEblue whose module membership >0.3 and gene significance for mRNAsi >0.3 (Figure 3(d)).

### 3.3. Identification of OS-Associated SRGs

**Figure 4:**
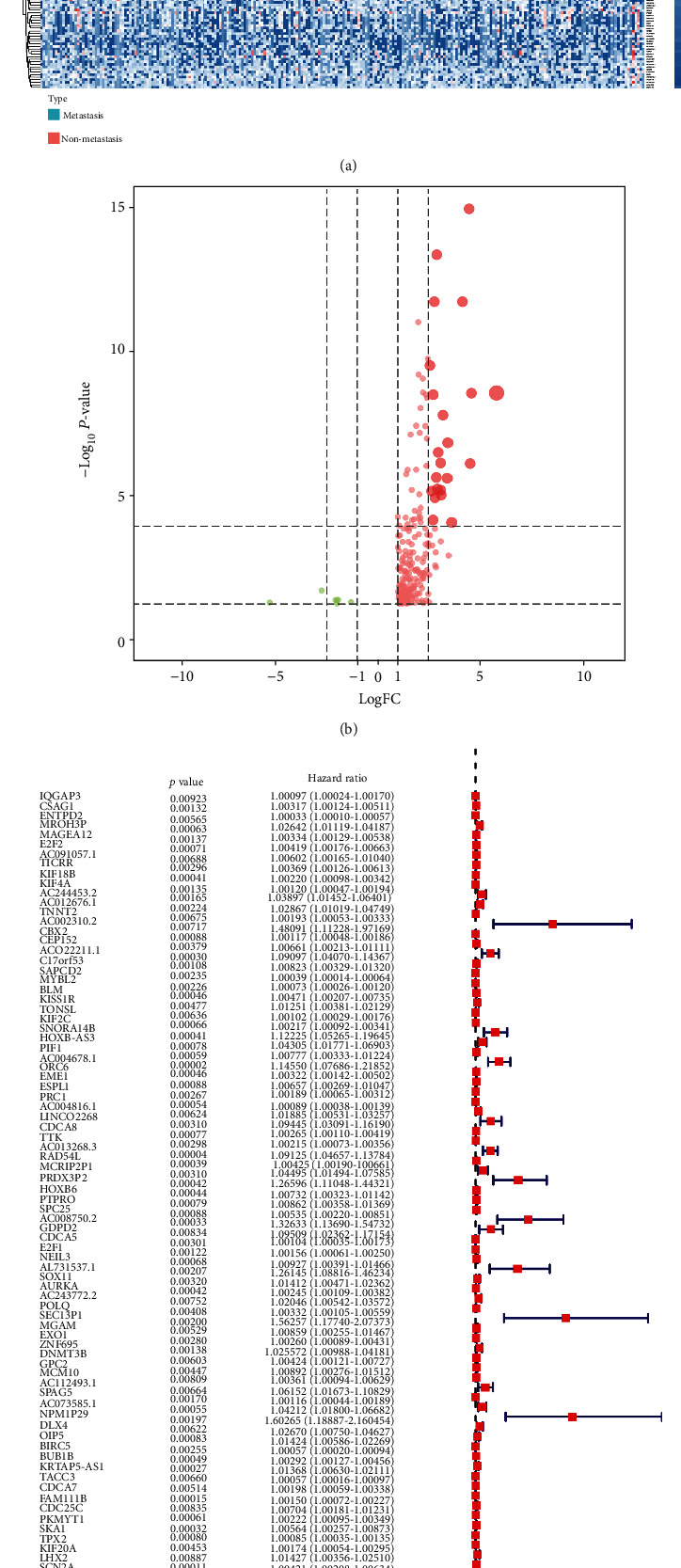
The identification of OS-related differentially expressed SRGs. The heat map (a) and volcano plot (b) of differentially expressed genes in MEblue module. The univariate regression analysis revealed the correlation of these differentially expressed SRGs with overall survival (c).

Those genes co-expressed significantly with the Egen of module (|Co-expression Coefficient| >0.50) and mRNAsi (|Co-expression Coefficient| >0.50) were extracted from MEblue module and identified as SRGs in the module. DEGs among these SRGs were illustrated in the heat map and volcano plot (Figures 4(a) and 4(b)). To assess the association between SRGs and overall survival (OS), the univariate Cox regression analysis was performed; the result of which was reported in the forest plot (Figure 4(c)).

### 3.4. The Construction of Prognostic Model Based on OS-Associated SRGs

**Figure 5:**
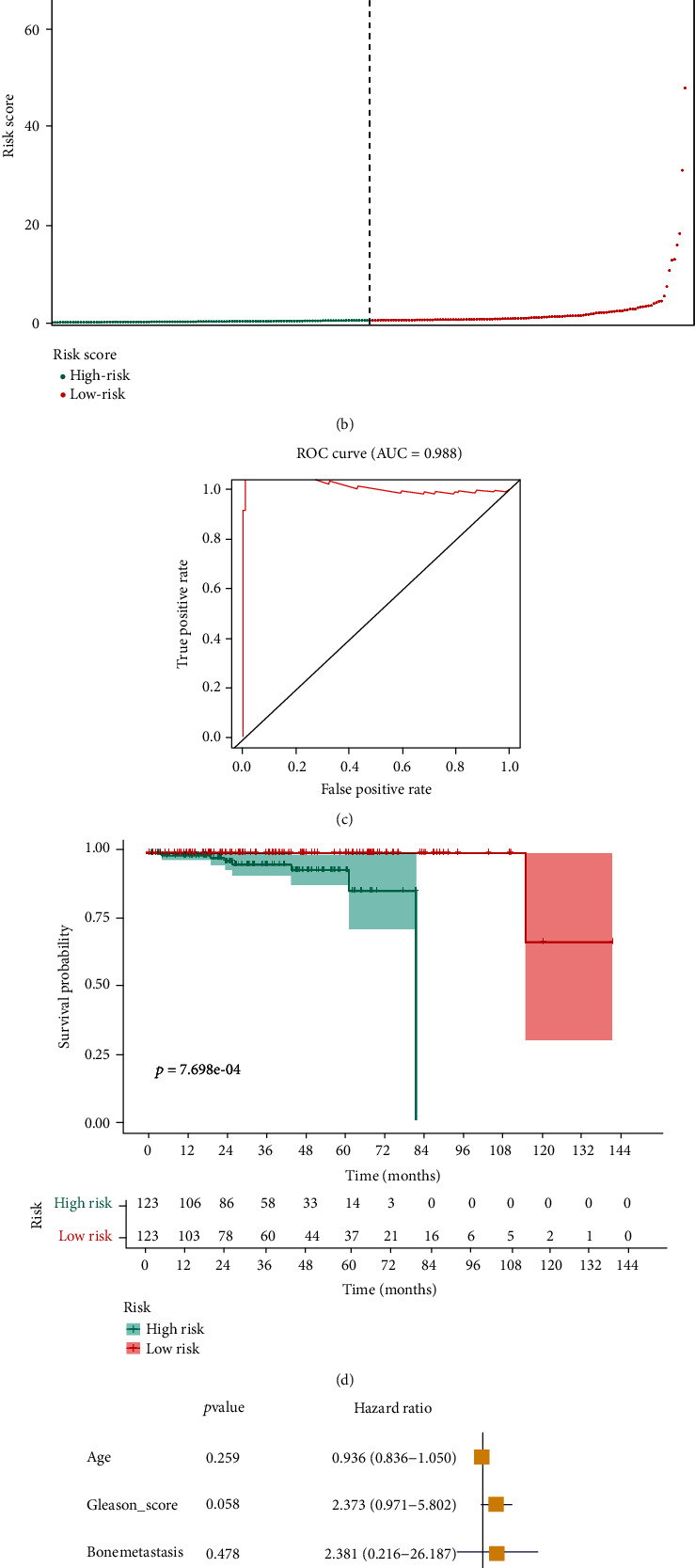
The establishment of prognostic model based on OS-related differentially expressed SRGs. The scatter plot (a) revealed the overall survival of high-risk and low-risk patients with PCa, with the risk curve (b) indicating the risk score of these patients. The ROC curve (c) validated the efficacy of this model (AUC =0.988). The Kaplan-Meier analysis between the high-risk and low-risk groups (d). The risk score of the prediction model was also proved to be an independent prediction factor by univariate cox regression analysis (HR =146.682, *p* < 0.001) and multivariate cox regression analysis (HR =1.055, *p* = 0.002) (e, f).

In order to further evaluate the prognostic value of SRGs, the multivariate Cox regression analysis was applied to establish a prognosis model, helping to predict the overall survival of PCa patients. The accuracy of the model was proved by area under ROC (AUC) curve (AUC =0.988) (Figure 5(c)). The Kaplan-Meier plot validated that statistical difference existed in overall survival between high-risk group and low-risk group (*p* < 0.001) (Figure 5(d)). Besides, the risk score and survival situation of patients with PCa were illustrated in the risk curve and scatter plot (Figures 5(a) and 5(b)). The results indicated that PCa patients with low risk were more likely to have a longer life expectancy than PCa patients with high risk. In addition, the risk score was identified as an independent prognosis factor by univariate cox regression analysis (HR =146.682, *p* < 0.001) and multivariate cox regression analysis (HR =1.055, *p* = 0.002).

### 3.5. The Identification of Underlying Pathway Mechanism

**Figure 6:**
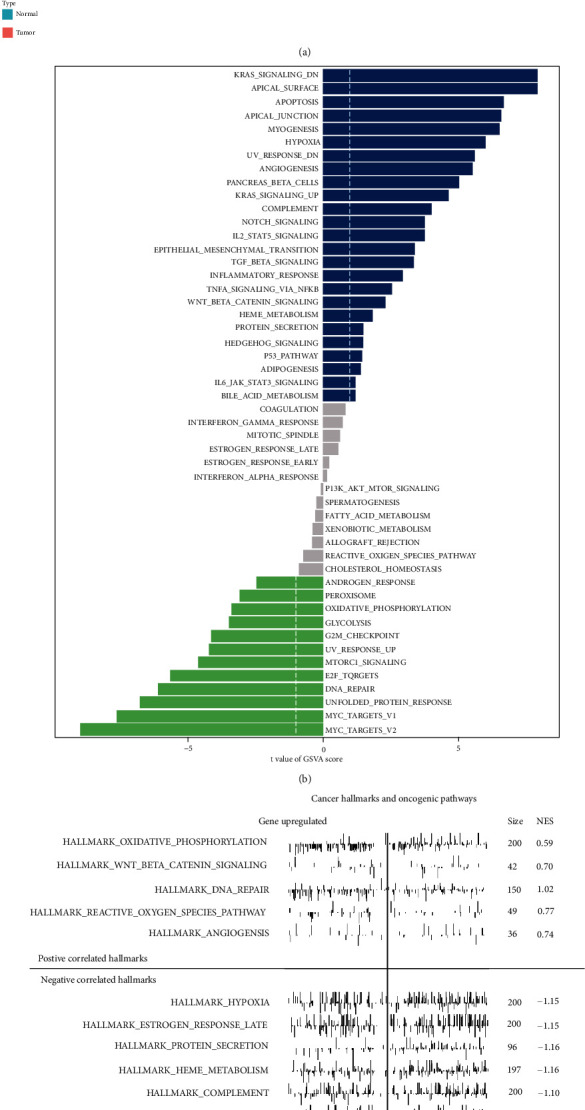
The exploration of the downstream pathway mechanism. The heat map revealed the differentially expressed hallmarks of cancer between normal tissue and PCa tissue (a) in GSVA analysis, with the *t* value shown in the bar chart (b). The upregulated and downregulated hallmarks of cancer were identified in GSEA analysis (c).

The heat map revealed the differentially expressed hallmarks of cancer between normal and tumor tissue in GSVA analysis (Figure 6(a)), whose *t* value of GSVA score was reported in the bar chart (Figure 6(b)). In addition, GSEA plot indicated positive correlated hallmarks of cancer (gene upregulated) and negative correlated hallmarks (genes downregulated) (Figure 6(c)). The Venn plot revealed that there were 11 hallmarks of cancer reported in both GSVA and GSEA analyses, which were considered the potential pathway mechanisms of PCa development.

### 3.6. The Regulation Pairs between TFs and Prognostic SRGs

**Figure 7:**
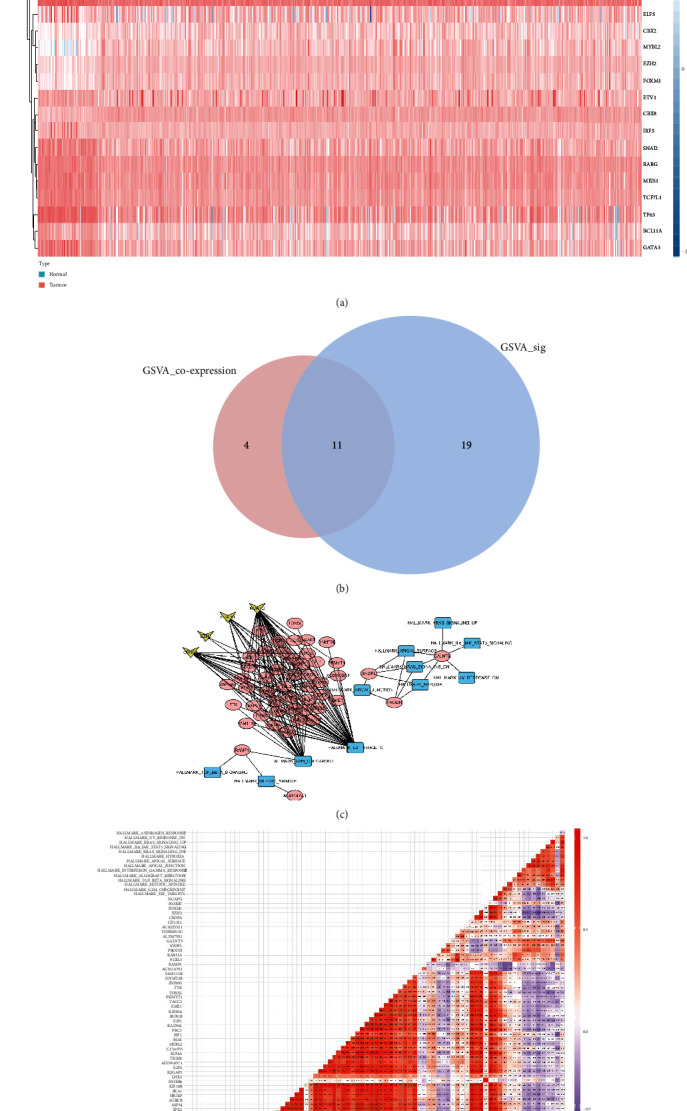
The regulation network among OS-related SRGs, transcription factors, and hallmarks of cancer. The heat map indicated differentially expressed cancer-associated transcription factors between normal and PCa tissues (a). The Venn plot indicated that a total of 11 hallmarks of cancer were differentially in both GSVA and GSEA analyses (b). The network revealed the interaction among OS-related SRGs, transcription factors, and hallmarks of cancer. Hallmark_ E2F_targets had the most prevalent connection with OS-SRGs. The heat map showed the correlation efficient among OS-related SRGs, transcription factors, and hallmarks of cancer in Pearson's correlation analysis. FOXM1 was significantly correlated with NEIL3 (correlation efficient =0.89, *p* < 0.001) and NEIL3 was significantly correlated with hallmark_E2F_targets (correlation efficient =0.58, *p* < 0.001).

The heat map revealed cancer-associated TFs differentially expressed between normal and PCa tissues (Figure 7(a)). The Pearson correlation analysis screened the regulation pair between differentially expressed cancer-associated TFs and prognostic SRGs, which all had a strong correlation (correlation coefficient>0.300，and *p* < 0.001). The interaction of these regulation pairs was indicated in the network (Figure 7(c)). Among 11 hallmarks found by GSVA and GSEA analyses, hallmark_E2F_targets had the most extensive interaction with SRGs, making it the most significant hallmark in PCa development and progression. The heat map revealed the correlation between the identified PCa-associated TFs, prognostic SRGs, and hallmarks of cancer. FOXM1 was significantly correlated with NEIL3 (correlation efficient =0.89, *p* < 0.001) and NEIL3 was significantly correlated with hallmark_E2F_targets (correlation efficient =0.58, *p* < 0.001). Finally, through comprehensive comparison and following experimental validation, FOXM1 regulated NEIL3 promoting hallmark_E2F_targets was considered a significant molecular mechanism of PCa development, progression, and metastasis.

### 3.7. Chromatin Immunoprecipitation Sequence (ChIP-seq) Validation

**Figure 8:**
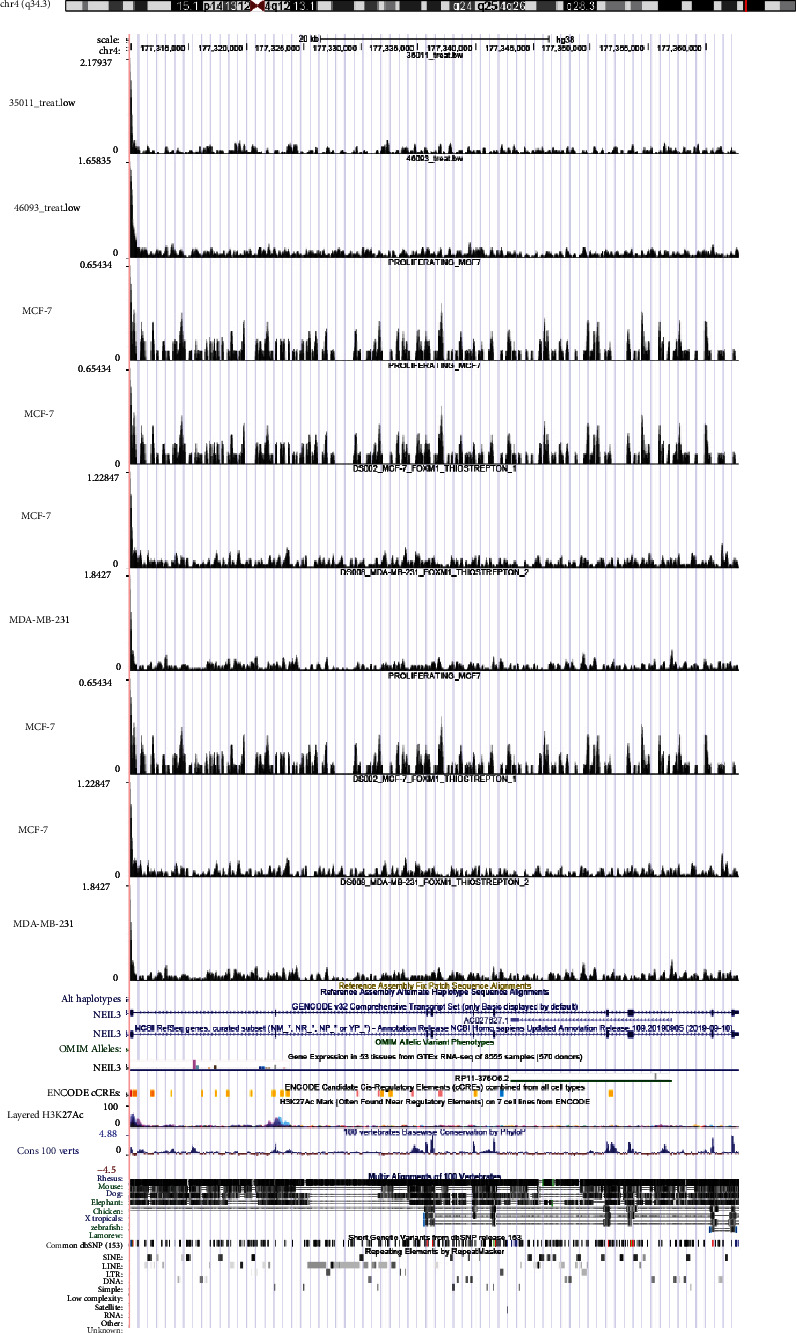
ChIP-seq validation. There existed several strong binding peaks of FOXM1 near the location of NEIL3 in samples of breast cancer.

Cistrome Data Browser is a public database for ChIP-seq data resources of humans and mouses. We acquired the ChIP-seq data of FOXM1 targeting NEIL3 in breast cancer from Cistrome Data Browser [31]. The results indicated that there were strong binding peaks of FOXM1 near the NEIL3 location in several samples, which provided the direct binding evidence of FOXM1 and NEIL3 (Figure 8).

### 3.8. Spatial Transcriptome and Single-Cell Sequencing

**Figure 9:**
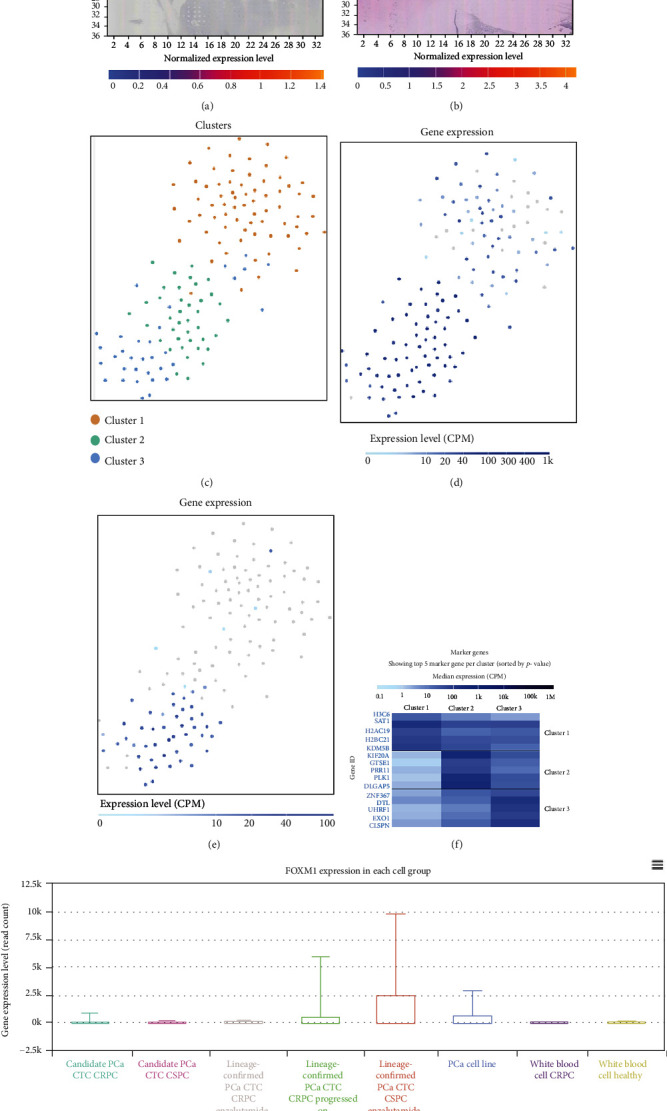
Spatial transcriptome and single-cell sequencing. The spatial transcriptome sequencing results indicating the location of FOXM1 and NEIL3 expression in prostate cancer tissue (Figures 9(a) and 9(b)). There were three clusters in the TSNE plot, which were untreated LNCap prostate cells after 0 hour (cluster 1), untreated LNCap prostate cells after 12 hours (cluster 2), and androgen treated LNCap prostate cells after 12 hours (cluster 3) (Figure 9(c)). FOXM1 and NEIL3 were mainly expressed in cluster 2 and cluster 3 (Figures 9(d) and 9(e)). The heat map of gene markers of each cluster (Figure 9(f)). FOXM1 was largely expressed in castration-sensitive prostate cancer (CSPC) circulating tumor cells (CTCs) and castration-resistant prostate cancer (CRPC) circulating tumor cells (CTCs) (Figure 9(g)).

The spatial transcriptome sequencing results acquired from SpatialDB showed the location of FOXM1 and NEIL3 expressions in prostate cancer (Figures 9(a) and 9(b)) [32]. Then, we downloaded the single-cell sequencing data from Single Cell Expression Atlas [17]. There were three clusters, which were untreated LNCap prostate cells after 0 hour (cluster 1), untreated LNCap prostate cells after 12 hours (cluster 2), and androgen-treated LNCap prostate cells after 12 hours (cluster 3), respectively (Figures 9(c) and 9(f)). Besides, FOXM1 and NEIL3 were mainly expressed in untreated LNCap prostate cells after 12 hours (cluster 2) and androgen-treated LNCap prostate cells after 12 hours (cluster 3) (Figures 9(d) and 9(e)). In addition, we also analyzed the expression of FOXM1 in single cell in the scRNASeq DB [17]. The results indicated that FOXM1 was largely expressed in castration-sensitive prostate cancer (CSPC) circulating tumor cells (CTCs) and castration-resistant prostate cancer (CRPC) circulating tumor cells (CTCs) (Figure 9(g)).

### 3.9. Specific Inhibitors of Biomarkers

**Figure 10:**
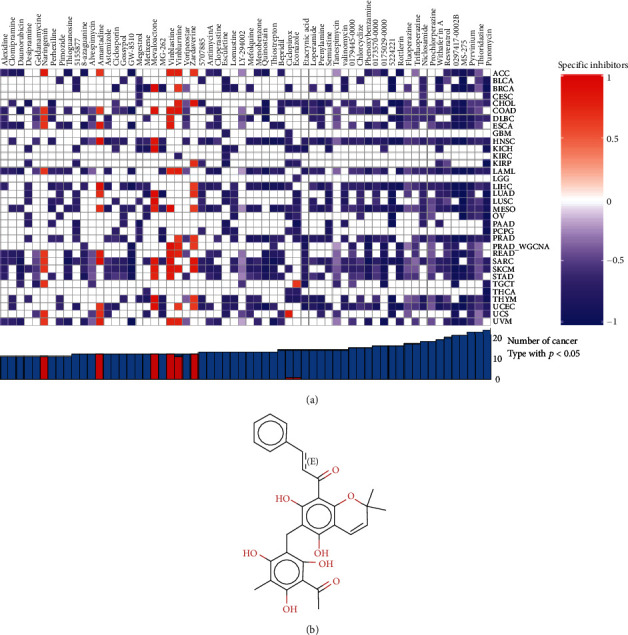
Specific inhibitors of key biomarkers. Potential specific small molecular inhibitors associated with stemness index among 33 types of cancers were shown in the heat map (a). The chemical structure rottlerin was acquired from the clue database.

In order to find possible target drugs or PCa, we applied CMap algorithm to identify specific inhibitors of key molecules in the regulatory axis and compared the correlation between these identified inhibitors and mRNAsi among 33 types of cancers. The results indicated that rottlerin was correlated with PCa mRNAsi, considered a potential specific inhibitor for PCa (Figure 10(a)). Besides, the chemical structure of rottlerin was acquired from the clue database (Figure 10(b)).

### 3.10. External Validation

We utilized multiple online databases to minimize the bias and verify the reliability of our study. In the beginning, we found the biomarkers of hallmark_E2F targets in PathCards database, including SRSF2, HELLS, PNN, TK1, and CKS2. UALCAN and UCSC xena database both indicated that the expression of FOXM1, NEIL3, SRSF2, HELLS, PNN, TK1, and CKS2 was correlated with PCa metastasis (*p* < 0.001) (Figure S1, S4). Besides, the expression of FOXM1, NEIL3, SRSF2, HELLS, PNN, TK1, and CKS2 was proved to significantly correlate with overall survival in GEPIA, UALCAN, and cBioPortal database (Figure S1, S2, S3). GEPIA and cBioPortal database validated that FOXM1, SRSF2, HELLS, PNN, TK1, and CKS2 were significantly co-expressed with NEIL3 (Figure S1, S3). In addition, the expression of these biomarkers in tissue level was detected in The Human Protein Atlas (Figure S5). Oncomine, firebrowse, and CCLE database revealed the expression of biomarkers in multiple cancer cell lines (Figure S6, S7, S8). The interaction plot of FOXM1, NEIL3, and genes of hallmark_E2F_targets was generated by STRING database (Figure S9).

## 4. Discussion

As the second common cancer in adult man, prostate cancer is one of the major causes for the death of patients with cancer. Despite the achievement of people's understanding of PCa, the lack of effective molecular targets makes it hard to handle the high probability of metastasis and mortality [1]. Primary treatments, such as surgery, radiotherapy, and active surveillance, could influence patients' functional and psychological conditions to some extent [4]. With ADT recommended for symptomatic patients with metastasis, the resistance for castration also bothers patients and oncologists, demanding for new therapeutic targets for mCRPC [8]. Recently, cancer stem cells (CSC) have been attached much importance in the aspects of cancer tumorigenesis, recurrence, and metastasis. Therefore, stemness indices were applied to evaluate tumor development, including mDNAsi and mRNAsi [33]. However, stemness-related genes in PCa were still lacking relevant studies.

In this study, we downloaded RNA-seq information of 406 patients with PCa from TCGA database. And we identified 538 DEGs between localized and metastatic PCa. Based on mRNAsi of PCa samples in TCGA database, we determined the gene module mostly correlated with mRNAsi using WGCNA. Then, we applied univariate and multivariate COX regression analyses to extract overall survival associated SRGs in the gene module. Furthermore, we discovered a significant regulatory pair of transcription factor (FOXM1) and OS-associated SRG (NEIL3) by Pearson's correlation analysis (*R* = 0.89，*p* < 0.001).

NEIL3, a DNA glycosylase in the Fpg/Nei family, carries out the first and most significant step of base excision repair (BER) though sundering bases damaged by reactive oxygen and inserting a DNA strand break [34]. Therefore, it is considered to function as a crucial safeguard to maintain the genome stability and evade mutations [35]. Recent studies have confirmed that the abnormal expression of NEIL3 was associated with the somatic mutation load in cancer [36]. The frequent mutation in NEIL3 in prostate cancer was also detected in Caucasians [37]. What's more, the variation of NEIL3 was found out to increase pesticide-associated prostate cancer risk after replication [38]. Although the underlying mechanism is still uncovered, NEIL3 can be considered a novel biomarker and potential therapy target for prostate cancer. In the present study, we identified NEIL3 as a significant prognostic SRG in PCa by univariate and multivariate regression analyses, and found out its significantly correlated transcription factor—FOXM1.

As one of the members of Forkhead family proteins, Forkhead Box M1 (FOXM1) functions as a transcriptional activator responsible for cell proliferation. The protein is phosphorylated in M phase and regulates the expression of many cell cycle genes, which are crucial for DNA replication and mitosis [39]. What is most attractive to us about FOXM1 is that it has been reported to overexpress in a wide range of cancers and contribute to all hallmarks of cancer [40]. Considered a potential oncogene and strong biomarker for multiple cancer, FOXM1 has emerged as an important molecule involved in tumorigenesis, development, and progression [41]. The suppression of tumor cell proliferation, migration, and angiogenesis has been observed when downregulating FOXM1 [42, 43]. The prognostic value of FOM1-regulatary network was also confirmed in 39 human malignancies [44]. Although the oncogenic mechanism of FOXM1 is still not fully understood, one of the main presumptions is that FOXM1 regulates the transcriptional activity of FOXM1 target genes, resulting in heighted cell proliferation and tumorigenic effects [45]. For example, the overexpression of FOXM1 synergistically with CENPF in prostate cancer leads to the activation of PI3K and MAPK signaling pathways, which are key signaling pathways involved in prostate cancer malignancy [46]. Besides, the dysregulation of FOXM1-CENPF due to miRNAs was discovered to contribute to the metastasis and drug resistance of prostate cancer [47].

Furthermore, we planned to discover the underlying mechanism of NEIL3 regulated by FOXM1 promoting PCa metastasis using GSVA and GSEA methods. The results indicated that hallmark_E2F_targets were differentially expressed between non-metastatic and metastatic PCa in both GSVA and GSEA analyses and significantly correlated with NEIL3 (*R* = 0.58，*p* < 0.001). Besides, we selected 5 key genes (SRSF2, HELLS, PNN, TK1, and CKS2) in hallmark_E2F_targets in Pathcards database and validated that they are associated with overall survival, metastasis, and tumor stage of patients with PCa through multiple online database validation. E2F transcription family is a large transcription family, all members of which contain one or more conservative DNA binding domains (DBDs). They play a crucial role in cell proliferation and cell apoptosis, and involve in the formation, development, and progression of multiple malignancies [48, 49]. E2Fs regulate the cell cycle transiting from G1 phase to S phase by interacting with plenty of cyclin-dependent proteins and kinases like Rb. Many researchers reported that the loss of RB protein function due to mutation causes the dysregulation of cell proliferation by overacting E2F, thus leading to oncogenic effect [50, 51]. The carcinogenic effect of E2F has been observed in many malignant tumors. For instance, the overexpression of E2F1, E2F2, and E2F3 was observed in patients with hepatocellular cancer, bladder cancer, retinoblastoma, and liposarcoma [52–54]. It is also reported that knockdown of E2F1 inhibited tumor cell proliferation and promoted apoptosis in castration-resistant prostate cancer (CRPC) by regulating TMOD2 and AIF1L expression [55, 56].

In order to explore potential inhibitor of PCa and validate the value of FOXM1-NEIL3 regulatory axis as therapy target, we utilized CMap algorithm to uncover small molecular inhibitors of the axis. Through comprehensive comparison among 33 cancers, we discovered that rottlerin might function as an effective specific drug for PCa. Previous studies have initially reported the suppressive effect of rottlerin on prostate cancer [57], although the underlying mechanism is still unclear. Rottlerin was reported to promote cell apoptosis by regulating ATM phosphorylation, which regulates FOXM1 through E2F [58, 59]. What's more, rottlerin and NEIL3 both get involved in cell damage repair [58], indicating the potential regulation between rottlerin and NEIL3. More in vivo and in vitro evidence are needed to validate the effectiveness of rottlerin as inhibitor.

As the first-line treatment for patients with PCa, androgen deprivation therapy (ADT) is often faced with the dilemma caused by CRPC [60]. Although novel hormone therapies were shown to improve OS in high-risk M0 CRPC, the chemotherapies for metastatic CRPC did not achieve satisfactory overall survival improvement [61]. However, the mechanism of the shift from androgen-dependent (AD) to androgen-independent (AI) cancer is still largely unknown [62]. The role of chronic inflammation in prostate cancer has been attached too much importance recently, due to its disruption to immune reaction and tumor microenvironment [63]. IL-6, a biomarker of prostate inflammation, was reported to contribute to the transition to CRRC through accessory activation of androgen receptors [64]. Furthermore, inflammatory indices have shown stratification efficacy in predicting prognosis of mCRPC patients treated with radiation [65]. Besides, the reduction of molecular biomarkers, including CD34 and VEGF, was observed in patients treated with 5*α*-reductase inhibitor before bipolar transurethral resection of the prostate (B-TURP) [66].

Previous studies have revealed that FOXM1 was a main driver for castration-resistant prostate cancer (CRPC) [67]. Besides, the expression of FOXM1 was promoted by SETD1A, which was expressed higher in CPRC than primary prostate cancer and facilitated stem cell factors and stem cell formation in metastatic CRPC [68]. In our study, we utilized single-cell sequencing and found out that FOXM1 was highly expressed in CRPC. Therefore, we are curious about the role of FOXM1 in CRPC formation and planned to carry out relevant studies to explore its underlying mechanism.

Although many methods have been taken to reduce possible bias in bioinformatics analysis, there were still some weaknesses in this study. Firstly, PCa patients acquired from the TCGA database were mainly from western countries, lacking enough data of Asian people. Besides, although we have validated the gene and protein expression levels of key biomarkers at the tissue and molecular levels in multiple databases (Figure S1-S9), this study was a correlation study from multiple dimensions instead of a direct biological mechanism study. However, this study did firstly identify OS-associated SRGs in PCa and construct a reliable model to predict survival of PCa patients. More importantly, we proposed a hypothesis that the mechanism of NEIL3 regulated by FOXM1 might play an important role in PCa stemness and promote PCa metastasis. The specific drug for the regulatory axis was also identified as rottlerin, providing new sights for PCa treatment. Importantly, to further explore the molecular mechanism and validate our hypothesis, we will perform rigorous cell, animal experiment, and clinical trials in the future.

## Data Availability

The datasets generated and/or analyzed during the current study are available in the TCGA-PCa program (https://portal.gdc.cancer.gov).

## Supplementary Materials

Supplementary Materials Table S1 The baseline information of PCa patients obtained from TCGA database. Table S2 Summary of multidimensional external validation results based on multiple databases. Figure S1 Oncomine database validation genes including FOXM1 (A), NEIL3 (B), HELLS (C), PNN (D), TK1 (E), and CKS2 (F) were overexpressed in PCa tissues. Figure S2 The Human Protein Atlas validation. The immunohistochemical results showed that the protein expression level of SRSF2 (B), HELLS (C), PNN (D), and TK1 (E) in PCa tissue was significantly higher than that in normal tissue. Figure S3 firebrowse database validation. The expression of FOXM1 (A), NEIL3 (B), TK1 (C), HELLS (D), CKS2 (E), and PNN (F) in PCa tissue was higher than that in normal tissue. Figure S4 CCLE database validation. mRNA expression of FOXM1 (A), HELLS (D), PNN (E), and TK1 (F) was higher than that in other cancer cell lines, while mRNA expression of NEIL3 (B), SRSF2 (C), and CKS2 (G) was lower than that in other cancer call lines. Figure S5 GEPIA database validation. The expression of FOXM1 (A), NEIL3 (B), TK1 (F), and CKS2 (G) was higher in PCa tissue, while the expression of SRSF2 (C), HELLS (D), PNN (E) was higher in normal tissue. Besides, the expression of SRSF2 (*p* = 0.021) (H), HELLS (*p* = 0.027) (I), PNN (*p* = 0.038) (J), TK1 (*p* = 0.046) (K), and CKS2 (*p* = 0.048) (L) was significantly with overall survival. The expression of FOXM1 (*R* =0.84, *p* < 0.001) (M), SRSF2 (*R* =0.33, *p* < 0.001) (N), PNN (*R* =0.33, *p* < 0.001) (O), TK1 (*R* =0.75, *p* < 0.001) (P), HELLS (*R* =0.66, p<0.001) (Q), and CKS2 (*R* = 0.7, *p* < 0.001) (R) was also validated to significantly with the expression of NEIL3. Figure S6 UALCAN database validation. The expression of FOXM1 (A), NEIL3 (B), HELLS (C), PNN (D), TK1 (E), and CKS2 (F) was higher in PCa tissue than that in normal tissue. Besides, the expression of FOXM1 (A), NEIL3 (B), HELLS (C), PNN (D), TK1 (E), and CKS2 (F) was all significantly correlated with tumor stage (*p* < 0.001). The expression of NEIL3 (*p* = 0.0049) (B), HELLS (*p* = 0.016) (C), PNN (*p* = 0.0055) (D), and CKS2 (*p* = 0.019) (F) was significantly with overall survival. Figure S7 cBioPortal database validation. These biomarkers' overall survival and mRNA expression *Z*-score were shown in the heat map (A). The overall expression of 7 integrated genes was significantly correlated with PCa patients' overall survival (*p* = 0.029) (B). Besides, the expression of SRSF2 (*p* = 0.0236) (C), PNN (*p* < 0.001) (D) was associated with overall survival. The expression of FOXM1 (*R* = 0.81, *p* < 0.001) (E), HELLS (*R* =0.66, *p* < 0.001) (F), PNN (*R* = 0.16, *p* < 0.001) (G), TK1 (*R* = 0.70, *p* < 0.001) (H), CKS2 (*R* = 0.48, *p* < 0.001) (I), SRSF2(*R* = 0.19, *p* < 0.001) (J) was significantly correlated with the expression of NEIL3. Figure S8 UCSC Xena database validation. The expression level of 7 genes was shown in the heat map (A). The expression of FOXM1 (B), NEIL3 (C), SRSF2 (D), CKS2 (E), HELLS (F), PNN (G), and TK1 (H) was higher in tumor tissue and associated with metastasis. Figure S9 STRING database validation. The interaction network between FOXM1, NEIL3, and genes of hallmark_E2F_targets.
