# Supplementary material for: Construction of Bone Metastasis-Specific Regulation Network Based on Prognostic Stemness-Related Signatures in Prostate Cancer
Source: Dis Markers. 2022 Mar 29;2022:8495923. doi: 10.1155/2022/8495923 (PMC8983176; doi:10.1155/2022/8495923)
Supplement: Supplementary Materials — Table S1 The baseline information of PCa patients obtained from TCGA database. Table S2 Summary of multidimensional external validation results based on multiple databases. Figure S1 Oncomine database validation genes including FOXM1 (A), NEIL3 (B), HELLS (C), PNN (D), TK1 (E), and CKS2 (F) were overexpressed in PCa tissues. Figure S2 The Human Protein Atlas validation. The immunohistochemical results showed that the protein expression level of SRSF2 (B), HELLS (C), PNN (D), and TK1 (E) in PCa tissue was significantly higher than that in normal tissue. Figure S3 firebrowse database validation. The expression of FOXM1 (A), NEIL3 (B), TK1 (C), HELLS (D), CKS2 (E), and PNN (F) in PCa tissue was higher than that in normal tissue. Figure S4 CCLE database validation. mRNA expression of FOXM1 (A), HELLS (D), PNN (E), and TK1 (F) was higher than that in other cancer cell lines, while mRNA expression of NEIL3 (B), SRSF2 (C), and CKS2 (G) was lower than that in other cancer call lines. Figure S5 GEPIA database validation. The expression of FOXM1 (A), NEIL3 (B), TK1 (F), and CKS2 (G) was higher in PCa tissue, while the expression of SRSF2 (C), HELLS (D), PNN (E) was higher in normal tissue. Besides, the expression of SRSF2 (p = 0.021) (H), HELLS (p = 0.027) (I), PNN (p = 0.038) (J), TK1 (p = 0.046) (K), and CKS2 (p = 0.048) (L) was significantly with overall survival. The expression of FOXM1 (R =0.84, p < 0.001) (M), SRSF2 (R =0.33, p < 0.001) (N), PNN (R =0.33, p < 0.001) (O), TK1 (R =0.75, p < 0.001) (P), HELLS (R =0.66, p<0.001) (Q), and CKS2 (R = 0.7, p < 0.001) (R) was also validated to significantly with the expression of NEIL3. Figure S6 UALCAN database validation. The expression of FOXM1 (A), NEIL3 (B), HELLS (C), PNN (D), TK1 (E), and CKS2 (F) was higher in PCa tissue than that in normal tissue. Besides, the expression of FOXM1 (A), NEIL3 (B), HELLS (C), PNN (D), TK1 (E), and CKS2 (F) was all significantly correlated with tumor stage (p < 0.001). The expression of NEI [file 8495923.f1.docx]

**Supplementary materials**

**Table S1** the baseline information of PRAD patients obtained from TCGA database

| Variables | Total Patients (N = 406) |
| --- | --- |
| **Age, years** |  |
| Mean ± SD | 60.7 ± 6.96 |
| Median (Range) | 61 (41 - 78) |
| **Gleason Score** |  |
| 6 | 44 (10.84%) |
| 7  8  9  10 | 200 (49.26%)  56 (13.79%)  102 (25.12%)  4 (0.98%) |
| **Bone Metastasis** |  |
| 1 | 401 (98.77%) |
| 2 | 5 (1.23%) |
| **Distant metasatsis** |  |
| 1 | 339 (83.50%) |
| 2 | 67 (16.50%) |
| **New Tumor Event** |  |
| N1 | 332 (81.77%) |
| N2 | 74 (18.23%) |

**Table S2** Summary of multidimensional external validation results based on multiple databases

|  | FOXM1 | | NEIL3 | | SRSF2 | | HELLS | | | PNN | | TK1 | | | | CKS2 | | Results |
| --- | --- | --- | --- | --- | --- | --- | --- | --- | --- | --- | --- | --- | --- | --- | --- | --- | --- | --- |
|  | N | P | N | P | N | P | N | P | N | | P | | N | P | N | | P |  |
| Oncomine | ↓ | ↑ | ↓ | ↑ | NA | NA | ↓ | ↑ | ↓ | | ↑ | | ↓ | ↑ | ↓ | | ↑ | FOXM1, NEIL3, HELLS, PNN, TK1, and CKS2 were expressed higher in PRAD than in normal tissue. |
| The human protein atlas | - | - | NA | NA | ↓ | ↑ | ↓ | ↑ | ↓ | | ↑ | | ↓ | ↑ | - | | - | SRSF2, HELLS, PNN, and TK1were expressed higher in PRAD than in normal tissue. |
| firebrowse | ↓ | ↑ | ↓ | ↑ | NA | NA | ↓ | ↑ | ↓ | | ↑ | | ↓ | ↑ | ↓ | | ↑ | FOXM1, NEIL3, HELLS, PNN, TK1, and CKS2 were expressed higher in PRAD than in normal tissue. |
| GEPIA | ↓ | ↑ | ↓ | ↑ | ↑ | ↓ | ↑ | ↓ | ↑ | | ↓ | | ↓ | ↑ | ↓ | | ↑ | FOXM1, NEIL3, TK1, and CKS2 were expressed higher, while SRSF2, HELLS, PNN were expressed lower in PRAD than in normal tissue. |
| UALCAN | ↓ | ↑ | ↓ | ↑ | NA | NA | ↓ | ↑ | ↓ | | ↑ | | ↓ | ↑ | ↓ | | ↑ | FOXM1, NEIL3, HELLS, PNN, TK1, and CKS2 were expressed higher in PRAD than in normal tissue. |
| cbioportal | ↓ | ↑ | ↓ | ↑ | ↓ | ↑ | ↓ | ↑ | ↓ | | ↑ | | ↓ | ↑ | ↓ | | ↑ | FOXM1, NEIL3, SRSF2, HELLS, PNN, TK1, and CKS2 were expressed higher in PRAD than in normal tissue. |
| UCSC Xena | ↓ | ↑ | ↓ | ↑ | ↓ | ↑ | ↓ | ↑ | ↓ | | ↑ | | ↓ | ↑ | ↓ | | ↑ | FOXM1, NEIL3, SRSF2, HELLS, PNN, TK1, and CKS2 were expressed higher in PRAD than in normal tissue. |

Note: “N” was defined as normal; “P” was defined as Prostate adenocarcinoma;“↑” was defined as a significantly high-expressed gene; “↓” was defined as a significantly low-expressed gene; “NA” was defined as “Not available”; “-” was defined as a gene with no significant difference in expression.

Abbreviations: PRAD means Prostate adenocarcinoma; GEPIA, Gene Expression Profiling Interactive Analysis.


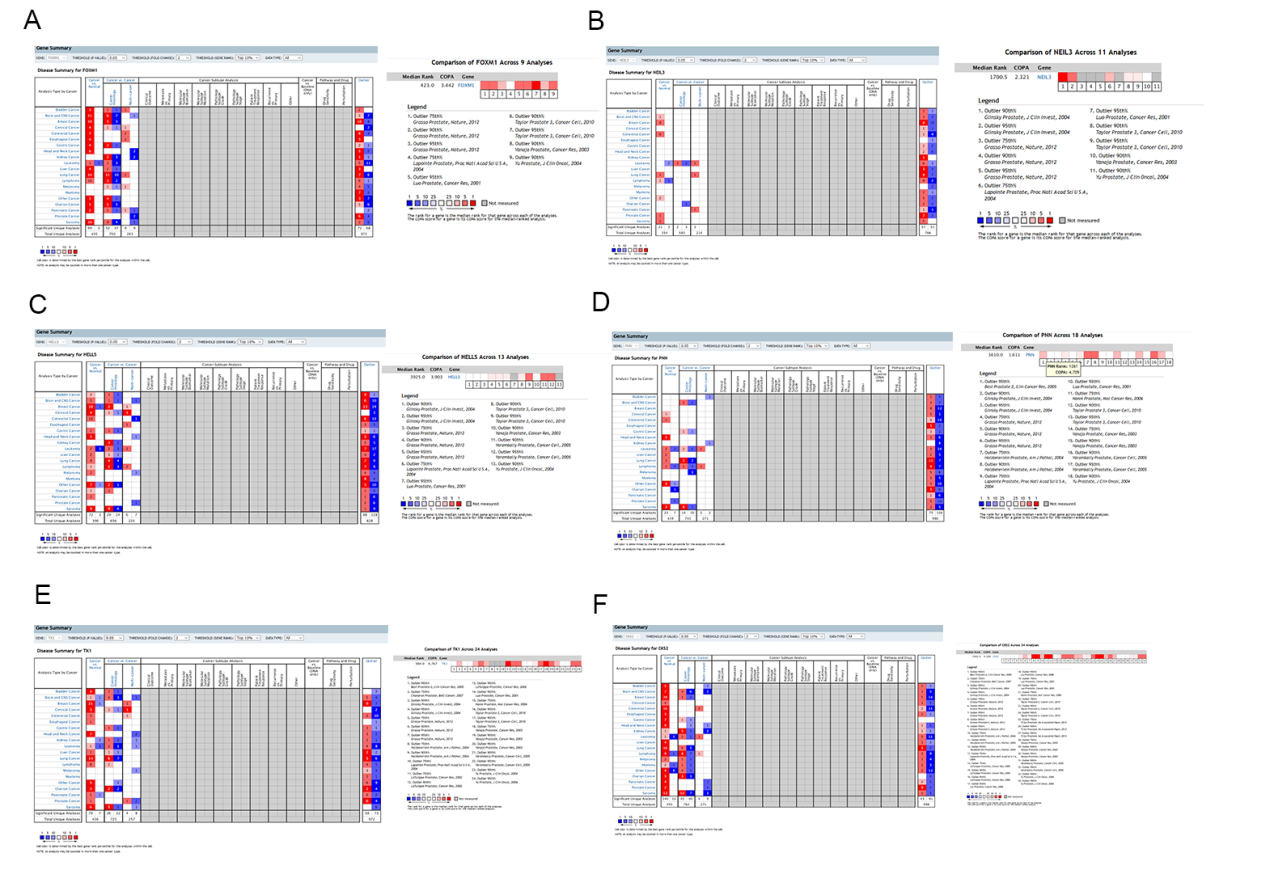


**Figure S1 Oncomine database validation**

Genes including FOXM1 (A), NEIL3 (B), HELLS (C), PNN (D), TK1 (E), and CKS2 (F) were over-expressed in PRAD tissues.


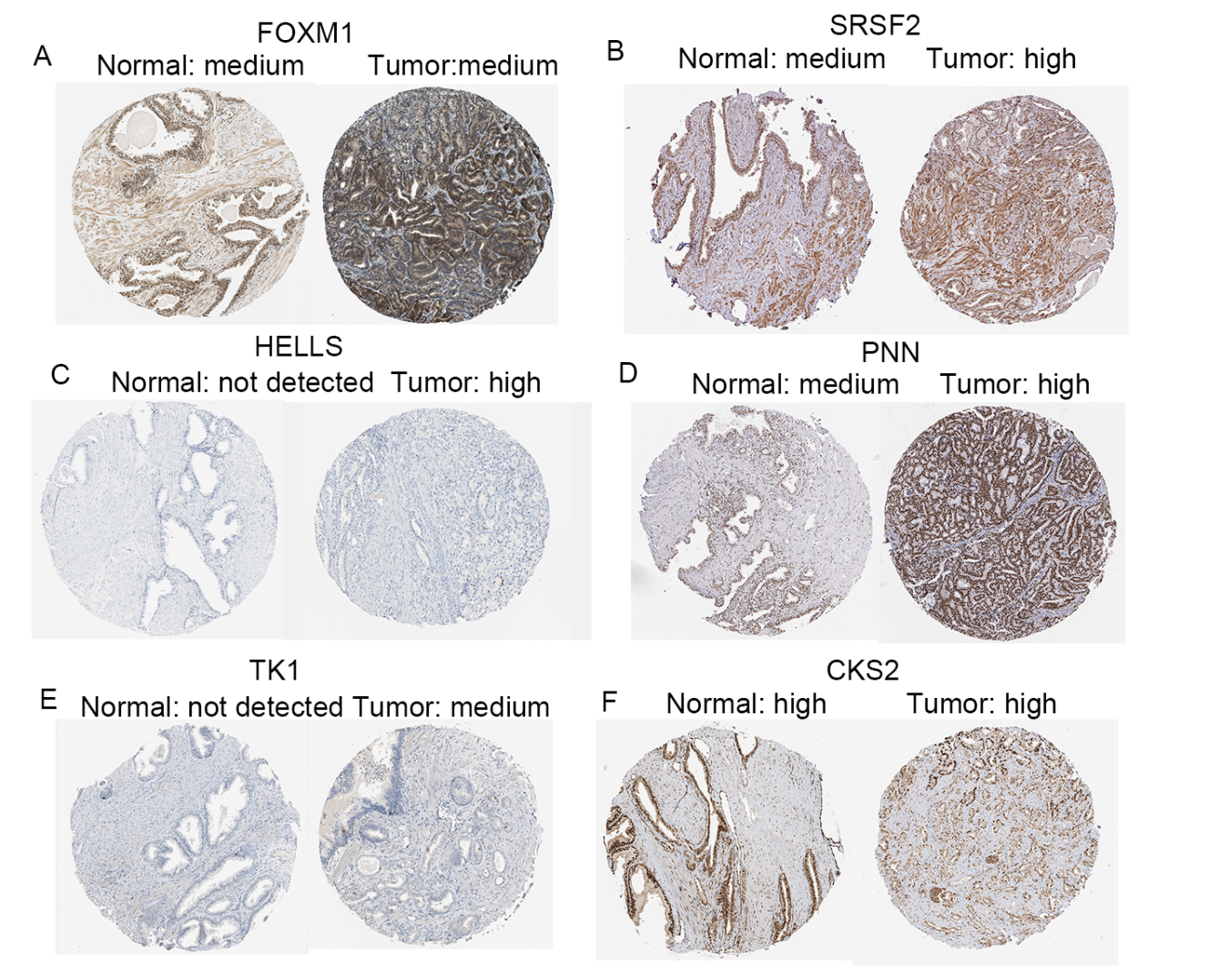


**Figure S2 the Human Protein Atlas validation**

The immunohistochemical results showed that the protein expression level of SRSF2 (B), HELLS (C), PNN (D), and TK1 (E) in PRAD tissue was significantly higher than that in normal tissue.


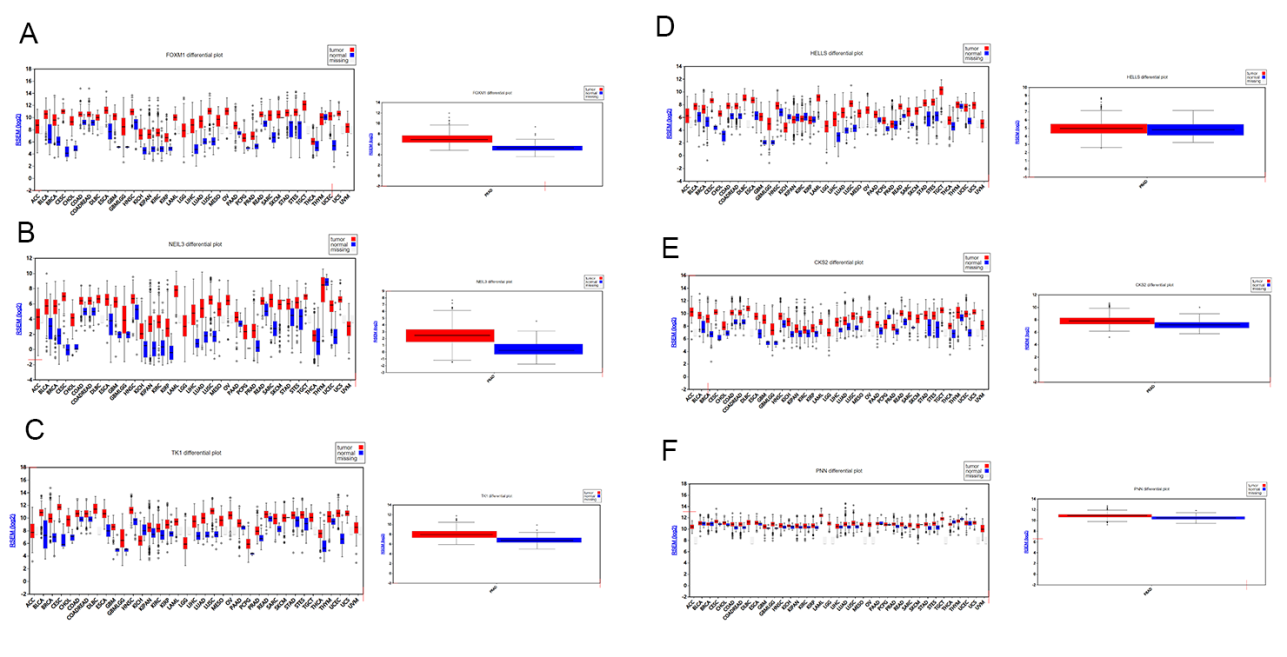


**Figure S3 firebrowse database validation**

The expression of FOXM1 (A), NEIL3 (B), TK1 (C), HELLS (D), CKS2 (E), and PNN (F) in PRAD tissue was higher than that in normal tissue.


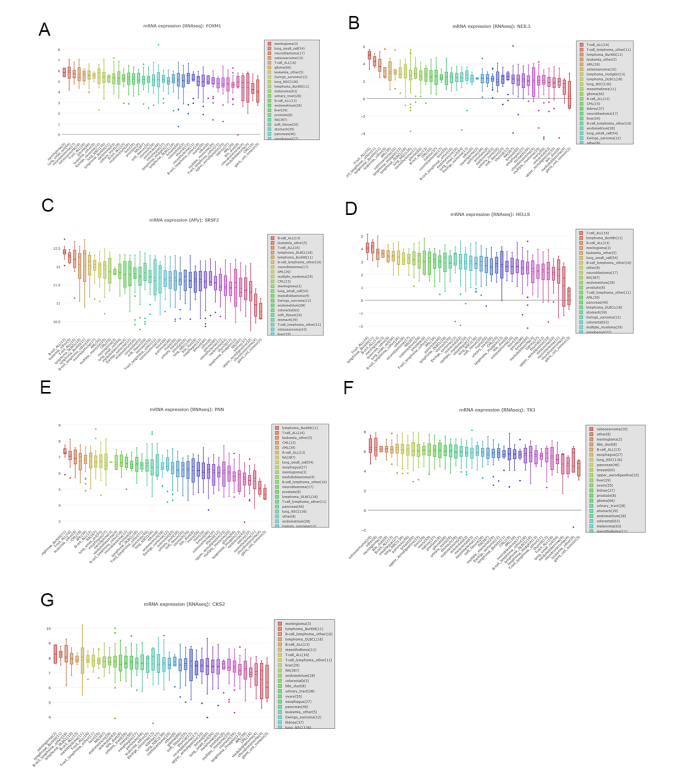


**Figure S4 CCLE database validation**

mRNA expression of FOXM1 (A), HELLS (D), PNN (E), and TK1 (F) was higher than that in other cancer cell lines, while mRNA expression of NEIL3 (B), SRSF2 (C), and CKS2 (G) was lower than that in other cancer call lines.


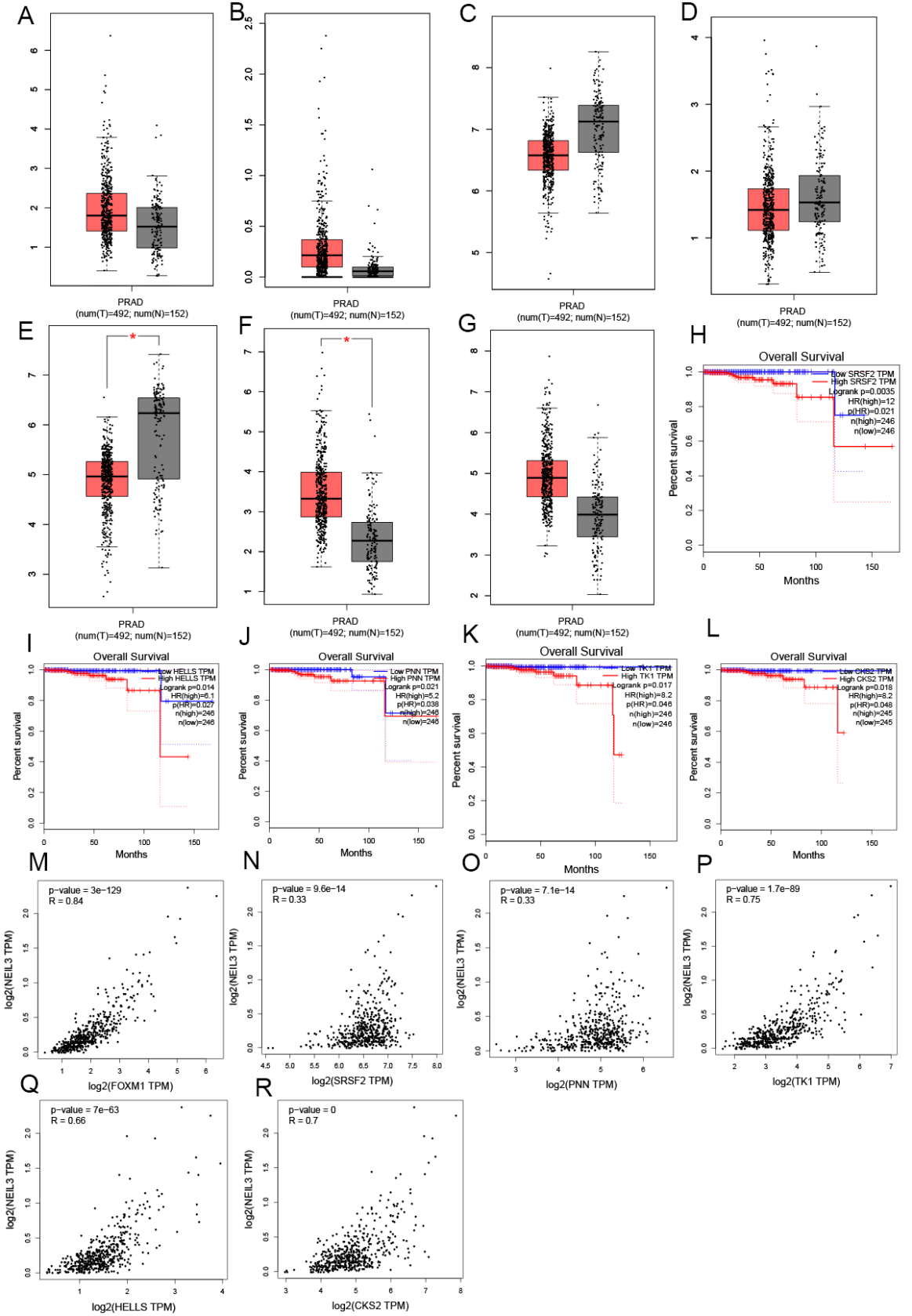


**Figure S5 GEPIA database validation**

The expression of FOXM1 (A), NEIL3 (B), TK1 (F), and CKS2 (G) was higher in PRAD tissue, while the expression of SRSF2 (C), HELLS (D), PNN (E) was higher in normal tissue. Besides, the expression of SRSF2 (p=0.021) (H), HELLS (p=0.027) (I), PNN (p=0.038) (J), TK1 (p=0.046) (K), CKS2 (p=0.048) (L) was significantly with overall survival. The expression of FOXM1 (R=0.84, p＜0.001) (M), SRSF2 (R=0.33, p＜0.001) (N), PNN (R=0.33, p＜0.001) (O), TK1 (R=0.75, p＜0.001) (P), HELLS (R=0.66, p＜0.001) (Q), CKS2 (R=0.7, p＜0.001) (R) was also validated to significantly with the expression of NEIL3.


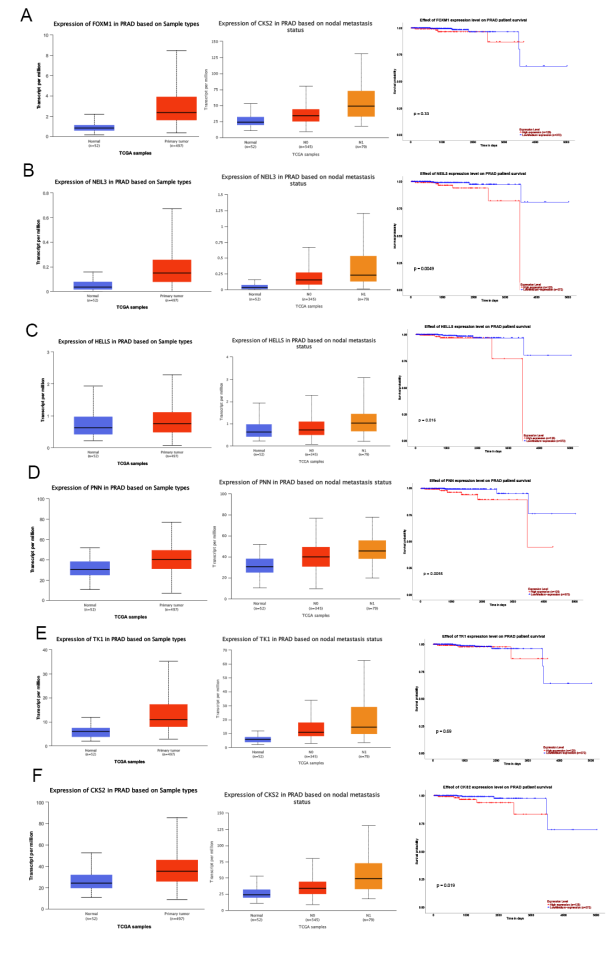


**Figure S6 UALCAN database validation**

The expression of FOXM1 (A), NEIL3 (B), HELLS (C), PNN (D), TK1 (E), and CKS2 (F) were higher in PRAD tissue that that in normal tissue. Besides, the expression of FOXM1 (A), NEIL3 (B), HELLS (C), PNN (D), TK1 (E), and CKS2 (F) was all significantly correlated with tumor stage (p＜0.001). The expression of NEIL3 (p=0.0049) (B), HELLS (p= 0.016) (C), PNN (p=0.0055) (D), and CKS2 (p= 0.019) (F) was significantly with overall survival.


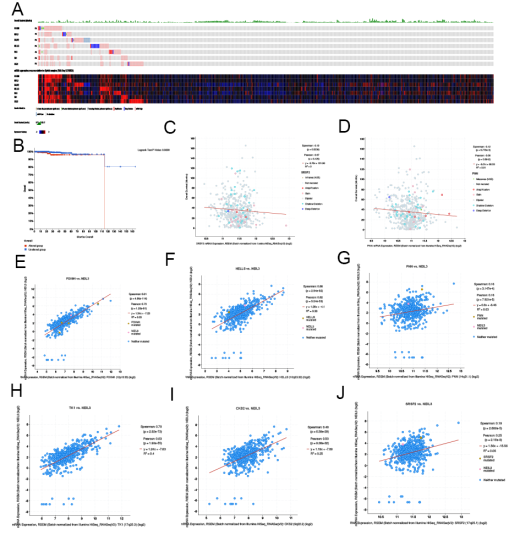


**Figure S7 cbioportal database validation**

These biomarkers’ overall survival and mRNA expression Z-score were shown in the heatmap (A). The overall expression of 7 integrated genes was significantly correlated with PRAD patients’ overall survival (P=0.029) (B). Besides, the expression of SRSF2 (p=0.0236) (C), PNN (p＜0.001) (D) was associated with overall survival. The expression of FOXM1 (R=0.81, p＜0.001) (E), HELLS (R=0.66, p＜0.001) (F), PNN (R=0.16, p＜0.001) (G), TK1 (R=0.70, p＜0.001) (H), CKS2 (R=0.48, p＜0.001) (I), SRSF2( R=0.19, p＜0.001) (J) was significantly correlated with the expression of NEIL3.


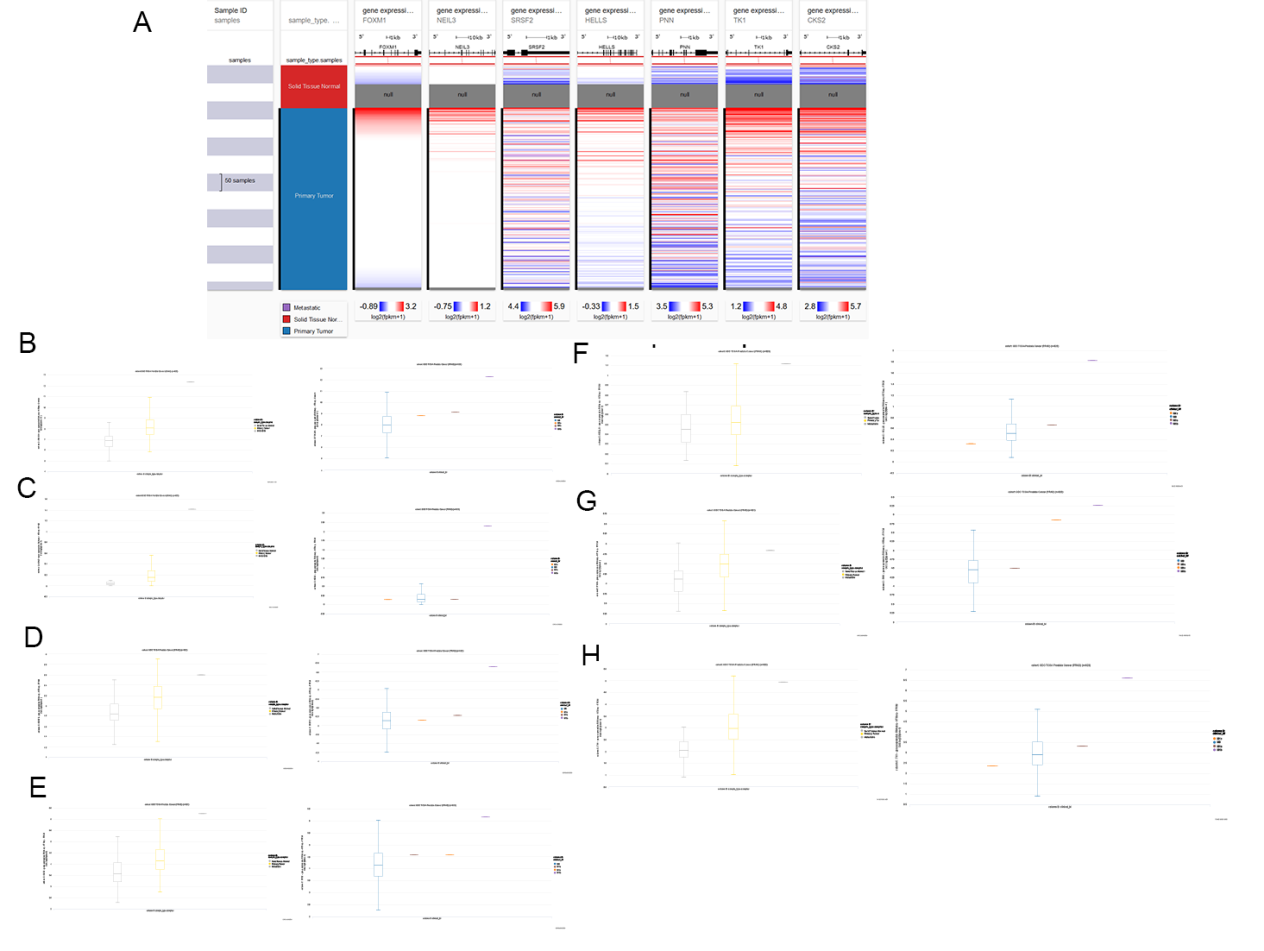


**Figure S8 UCSC Xena database validation**

The expression level of 7 genes was shown in the heatmap (A). The expression of FOXM1 (B), NEIL3 (C), SRSF2 (D), CKS2 (E), HELLS (F), PNN (G), TK1 (H) was higher in tumor tissue and associated with metastasis.


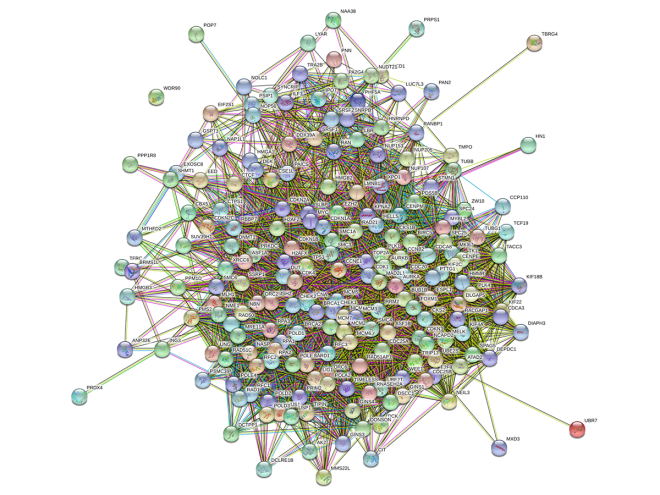


**Figure S9 STRING database validation**

The interaction network between FOXM1, NEIL3, and genes of hallmark_E2F_targets.
